# Acute cholecystitis in H-type duplicated gallbladder: a case report

**DOI:** 10.1093/jscr/rjaf720

**Published:** 2025-09-15

**Authors:** Nicolas Inga-Estrada, Martin Hemeryth-Rengifo, Fritz Fidel Váscones-Román, María Claudia Hinojosa-Ríos, Percy Amador Inga-San Bartolomé

**Affiliations:** Department of Medicine, Universidad Nacional de la Amazonía Peruana Loreto, Iquitos 16000, Peru; Astrocyte, Neurosurgical Research Group, Boston, MA, United States; Department of Medicine, Universidad Científica del Sur, Lima 15067, Peru; Astrocyte, Neurosurgical Research Group, Boston, MA, United States; Department of Medicine, Universidad Peruana Cayetano Heredia, Avenida Honorio Delgado 430, San Martín de Porres, Lima 15102, Peru; Instituto Nacional de Ciencias Neurológicas, Lima, Peru; Váscones’s Lab, Lima, Peru; Department of Medicine, Universidad Nacional de la Amazonía Peruana Loreto, Iquitos 16000, Peru; Department of Medicine, Universidad Nacional de la Amazonía Peruana Loreto, Iquitos 16000, Peru; Hospital Essalud III Loreto, Iquitos 16000, Peru

**Keywords:** cholecystectomy, laparoscopic, cholecystitis, gallbladder, magnetic resonance

## Abstract

Gallbladder duplication is a rare congenital anomaly. Abnormal biliary anatomy is associated with an increased risk of complications, such as bile duct injury, during cholecystectomy. In this article, we present a clinical case of gallbladder duplication identified preoperatively by magnetic resonance cholangiopancreatography, which guided surgical planning. A 70-year-old man was admitted with acute cholecystitis, low-grade fever, jaundice, and dyspnea. Magnetic resonance cholangiopancreatography revealed a duplicated gallbladder (Type H). During surgery, the chronically inflamed upper gallbladder was completely resected. The larger lower gallbladder, with acute cholecystitis and a Mirizzi-like pattern, was partially resected due to severe inflammation and the inability to identify the cystic duct. The patient had an uneventful course. Gallbladder duplication is a rare congenital anomaly that may be associated with other congenital anomalies. Thorough preoperative imaging studies, meticulous surgical technique, and rigorous intraoperative monitoring are essential, as these abnormalities can lead to serious injuries.

## Introduction

Duplicated gallbladder is a rare congenital anomaly, with an estimated prevalence of 1 to 5 per 10 000 individuals [1]. Autopsy studies and surgical series suggest a higher incidence in women, and most cases are diagnosed incidentally during surgical procedures, reoperations, or imaging studies performed for other indications [2–5].

Clinically, patients may remain asymptomatic or present with symptoms indistinguishable from biliary colic or acute cholecystitis [6, 7]. Gallstone disease is the most common clinical presentation [7].

Regarding imaging diagnosis, although abdominal ultrasound is the most commonly used initial modality, it has limitations in detecting this malformation. Magnetic resonance cholangiopancreatography (MRCP) is the imaging modality of choice, as it allows for confirmation of gallbladder duplication and accurate delineation of the biliary anatomy [8]. Preoperative identification of this anomaly is crucial, given its association with an increased risk of laparoscopic complications, such as vascular injuries or bile duct leaks [7, 9].

This article presents the case of a male patient with a preoperative diagnosis of duplicated gallbladder (H-type double gallbladder), confirmed intraoperatively during laparoscopic cholecystectomy, highlighting the diagnostic and surgical challenges associated with this rare entity.

## Case report

A 70-year-old man presented with 12 h of fever and malaise, followed by jaundice, right upper quadrant pain, and progressive dyspnea. Past history included Alzheimer’s disease, hypertension, hypothyroidism, and prior appendectomy.

**Figure 1:**
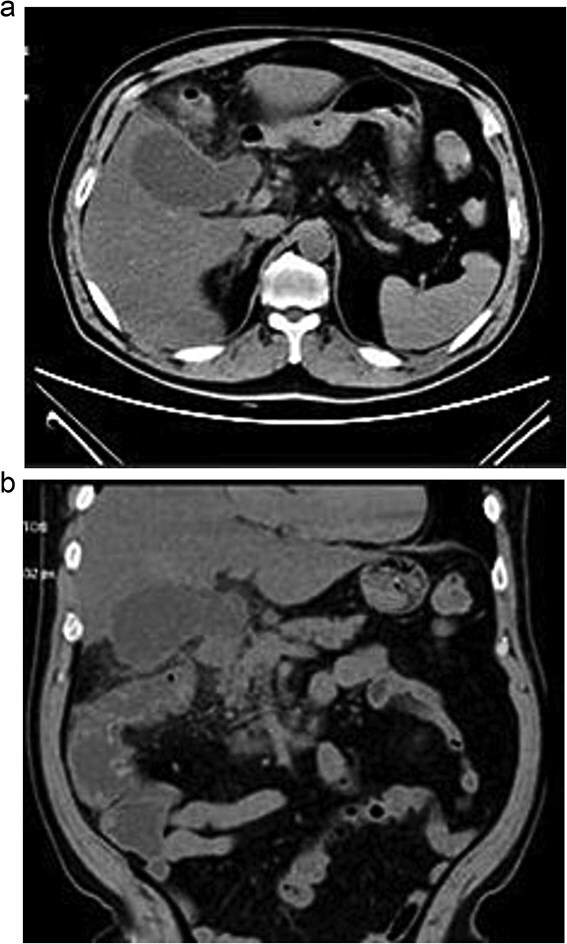
Axial (a) and coronal (b) abdominal CT showing gallbladder hydrocholecystosis.

**Figure 2:**
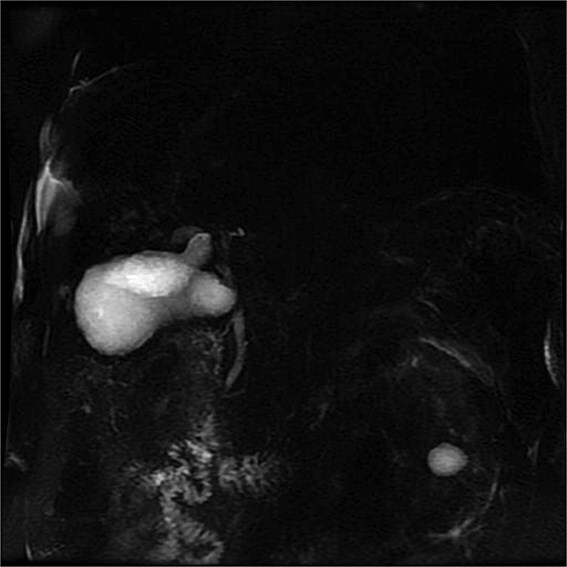
MRCP showing duplicated gallbladder (H-type), with two independent cystic ducts draining into the common bile duct.

**Figure 3:**
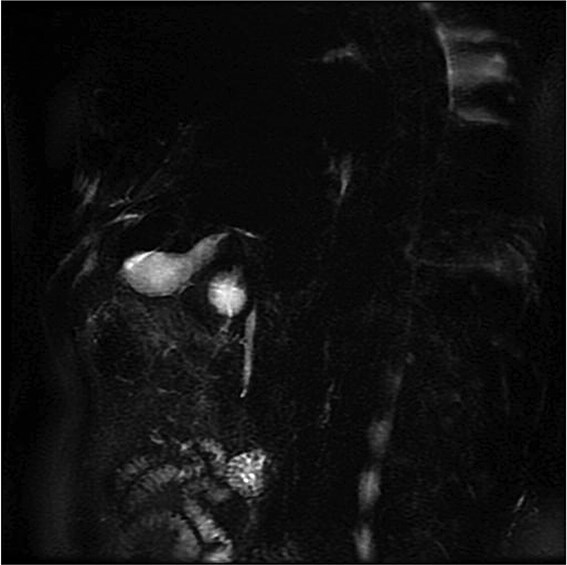
MRCP (thick-slab 2D coronal oblique) showing duplicated gallbladder (H-type) with two independent cystic ducts and no evidence of choledocholithiasis.

**Figure 4:**
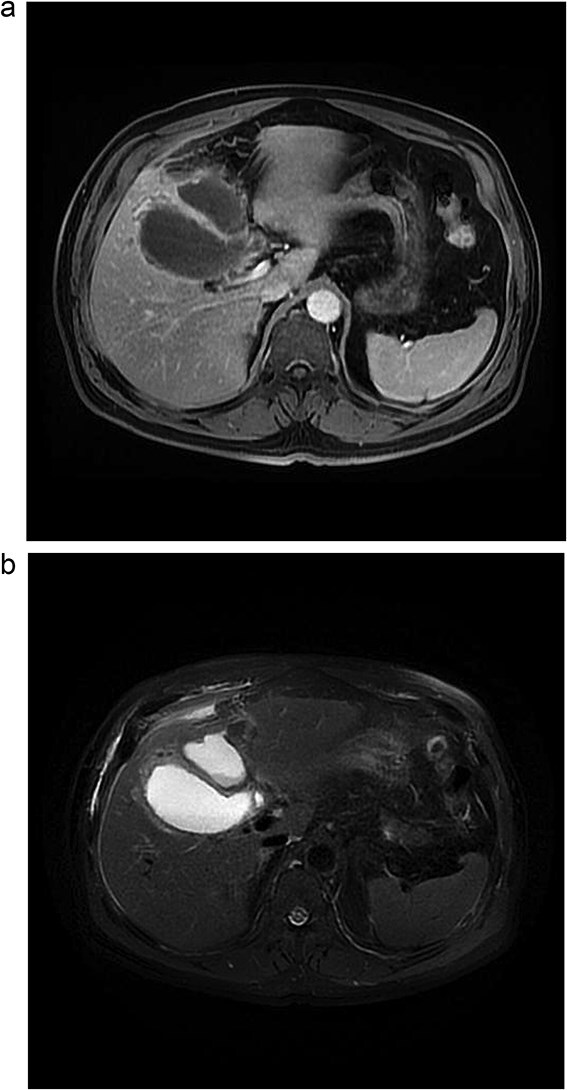
Abdominal MRI. (a) Axial 3D T1 LAVA-Flex *water-only* sequence, venous phase. (b) Axial T2 PROPELLER sequence with fat suppression.

Lab tests showed leukocytosis (15.2 × 10^3^/μl), thrombocytopenia (95.8 × 10^3^/μl), elevated CRP (25.22 mg/dl), procalcitonin (4.49 ng/ml), and total bilirubin (3.5 mg/dl). Liver function was otherwise normal. Ultrasound revealed lithiasic cholecystitis with hydrocholecystosis. Computed tomography (CT) failed to detect duplication (Fig. 1a and b). MRCP confirmed H-type duplicated gallbladder with two independent cystic ducts and mild extrinsic compression of the extrahepatic bile duct (Mirizzi-like pattern) (Figs 2 and 3). MRI sequences (T1 LAVA-Flex and T2 PROPELLER with fat suppression) also demonstrated the duplicated gallbladder and supported the MRCP findings (Fig. 4). No choledocholithiasis was seen.

After stabilization, laparoscopic cholecystectomy was performed. The superior gallbladder showed chronic inflammation; the inferior one had acute cholecystitis with necrosis and multiple stones. Both had independent cystic ducts. The superior gallbladder was fully resected after dissecting Calot’s triangle and clipping the cystic duct and artery. The inferior gallbladder had dense adhesions and necrosis; partial cholecystectomy was performed with resection of the anterior wall, careful hemostasis of the posterior wall, irrigation, and drain placement (Figs 6 and 7).

Both specimens were extracted in a bag. Histopathology revealed chronic cholecystitis with necrosis. The postoperative course was uneventful, and the patient was discharged in good condition.

**Figure 5:**
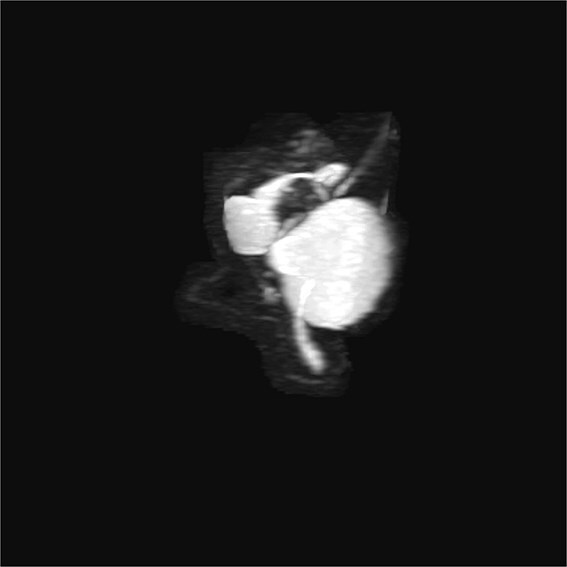
3D T2-weighted MRCP with maximum intensity projection (MIP), showing duplicated gallbladder (H-type) and biliary anatomy.

**Figure 6:**
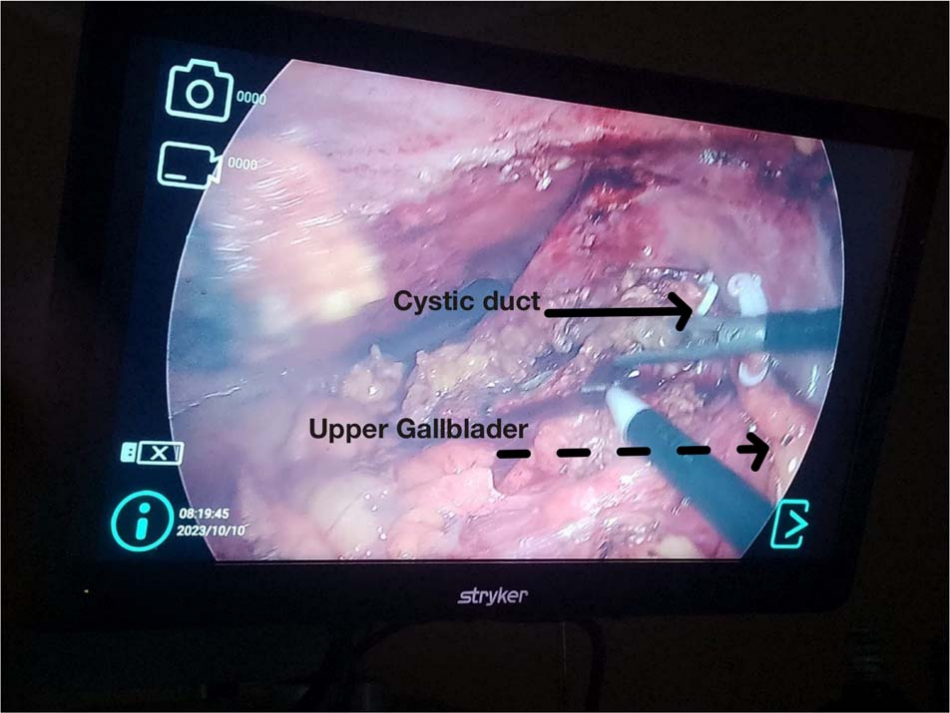
Intraoperative laparoscopic view. Solid arrow: Cystic duct with polymer clip. Dashed arrow: Upper gallbladder removed from its original position.

**Figure 7:**
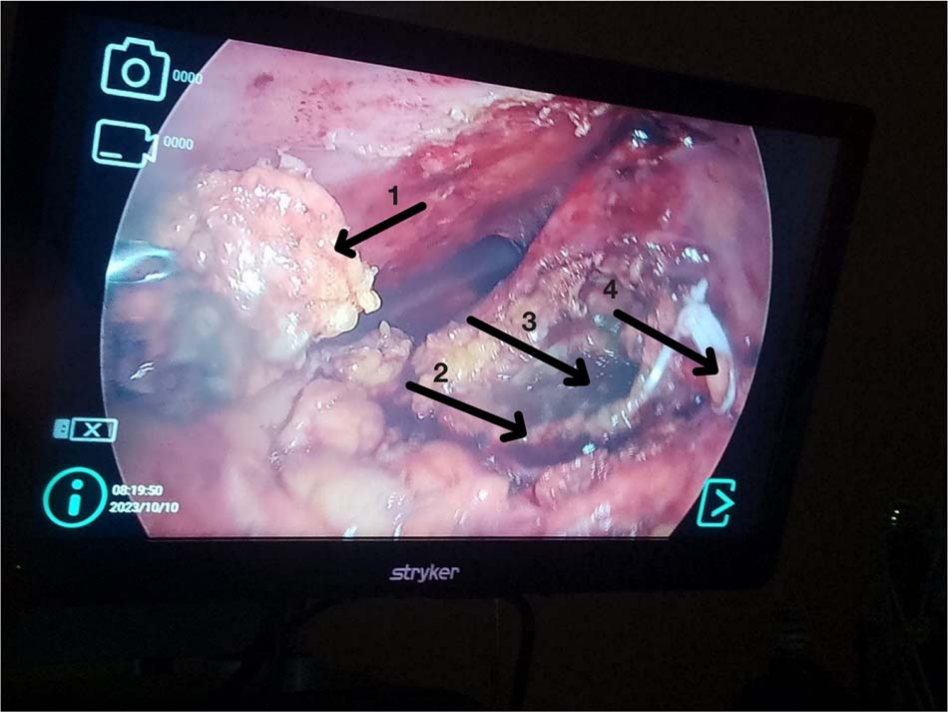
(1) Anterior wall of the inferior gallbladder. (2) Posterior wall of the inferior gallbladder. (3) Necrotic area of the posterior wall of the inferior gallbladder. (4) Upper gallbladder removed from its original position.

## Discussion

Duplication of the gallbladder arises from embryonic division of the hepatic diverticulum in early gestation. The Harlaftis classification defines Type IIA (H-type) as two gallbladders with separate cystic ducts draining into the common bile duct [10]. Though more frequent in women, it also occurs in men [7].

Most cases are diagnosed incidentally, but preoperative identification via MRCP is crucial in complex or recurrent biliary disease [8] (Figs 2–5). Anatomical variants increase the risk of bile duct injury, bleeding, or incomplete surgery [9]. Surgically, duplicated gallbladders pose technical challenges, especially when inflamed or fibrotic. Higher conversion and complication rates have been reported [7, 11].

In this case, chronic and acute inflammation coexisted. The superior gallbladder was resected completely. Severe inflammation in the inferior gallbladder precluded safe identification of the cystic duct; thus, a partial cholecystectomy was performed (Figs 6 and 7). This technique has been used successfully in complex cholecystitis cases [12]. Histology confirmed asynchronous inflammation. This emphasizes the clinical variability and surgical implications of gallbladder duplication. Surgeons must remain vigilant for this entity in patients with atypical symptoms or imaging.

## Conclusion

Duplication of the gallbladder is a rare congenital anomaly that may present significant diagnostic and surgical challenges. Surgeons should maintain a high index of suspicion for this condition during the patient’s preoperative evaluation, particularly in cases of atypical biliary disease. Careful preoperative imaging, meticulous surgical technique, and intraoperative vigilance are essential, as congenital anomalies such as duplicated gallbladder are critical predisposing factors for iatrogenic bile duct injuries and incomplete surgical treatment during cholecystectomy.
